# Prophylaxis and treatment of infection in long bones using an antibiotic-loaded ceramic coating with interlocking intramedullary nails

**DOI:** 10.5194/jbji-7-101-2022

**Published:** 2022-04-22

**Authors:** Emilie-Ann Downey, Kayla M. Jaime, Taylor J. Reif, Asim M. Makhdom, S. Robert Rozbruch, Austin T. Fragomen

**Affiliations:** 1 Department of Orthopaedics, Pierre-Boucher Hospital, Longueuil, QC, Canada; 2 Limb Lengthening and Complex Reconstruction Service, Hospital for Special Surgery, Weill Cornell Medicine, Cornell University, New York, NY, USA; 3 Department of Orthopaedics, King Abdulaziz University, Jeddah, Saudi Arabia

## Abstract

**Background**: The study was done (1) to report on our recent experience with antibiotic-loaded calcium sulfate-coated interlocking intramedullary nails (CS-IMN) for infection prevention or infection eradication and (2) to compare the efficacy of CS-IMN versus antibiotic-loaded polymethylmethacrylate-coated IMN (PMMA-IMN) for infection eradication. **Methods**: We retrospectively reviewed the medical records of consecutive patients who underwent a limb salvage procedure for infection cure or infection prevention with PMMA-IMN or CS-IMN. We reviewed patient demographics, host-type, pre-operative infecting organisms, intraoperative cultures, as well as our main outcomes: infection control rate, achievement of union/fusion, and limb salvage. **Results**: 33 patients were treated with CS-IMN: 9 patients with goal of infection cure and 24 patients for infection prophylaxis. When used for infection prophylaxis, there was a 100 % ( 24/24 patients) prevention of infection rate, 95.5 % union rate ( 21/22 patients), and 100 % ( 24/24 patients) limb salvage rate. Nine patients were treated with CS-IMN to eradicate infection and were compared to a cohort of 28 patients who were treated with PMMA-IMN. The infection was eradicated in 7/9 patients (77.8 %) in the CS-IMN group versus 21/26 patients (80 %) in the PMMA-IMN group ( p=0.44 ). Bone union/fusion was achieved in 8/9 patients (88.9 %) in the CS-IMN group versus 21/24 patients (87.5 %) in the PMMA-IMN group ( p=0.11 ). The limb salvage rate in the CS-IMN group was 100 % ( 9/9 patients) versus 89 % ( 25/28 patients) in the PMMA-IMN group. **Conclusions**: CS-IMN are safe and easy to use, and we have therefore expended our indications for them. CS-IMN are very effective at infection prophylaxis in high-risk cases where infection is suspected. Early analysis suggests that CS-IMN are non-inferior to PMMA-IMN for infection eradication. This is our preliminary data that show this novel technique to be safe in a small cohort and may be as effective as the more established method. Future studies with larger cohorts of patients will be required to confirm these findings.

## 1 Introduction

Chronic osteomyelitis is a devastating complication in orthopedic surgery. Osteomyelitis is difficult to treat and often complicated by the presence of internal implants at the infected site. The health status of the host, the condition of the local soft tissues, and the infective organism all influence the ability to eradicate the infection and achieve bony union. Antibiotic-loaded, polymethylmethacrylate-coated interlocking intramedullary nails (PMMA-IMN) have been well established for infection eradication (Conway et al., 2014; Makhdom et al., 2020). This technique allows for infection control with immediate stability of the bone through a single stage surgical intervention avoiding the need for external fixation. The locally active antibiotic carrier permits early and maintained effective local tissue antibiotic concentrations while also ensuring that systemic levels and associated toxicity remain low (Metsemakers et al., 2020).

Despite good clinical success, many practical limitations of PMMA-IMN remain. Because of the exothermic polymerization reaction that occurs during the curing process, heat stable antibiotics must be used. The cement mantle surrounding the IMN must be thick enough to withstand the stress of insertion into the IM canal without delaminating. Due to the space occupied by the cement, the bulk of the cement-coated nail increases significantly necessitating the use of a smaller diameter nail or excessive reaming, leading to less stable fixation. This method is not possible in narrow canals. The process required to create the PMMA-coated nail in the operating room is time-consuming and tedious. It may also be true that the presence of non-biodegradable PMMA cement leads to the need for a second surgery to remove the implant because of the long-term risk of bacterial adhesion and biofilm formation (Neut et al., 2003). Finally, the mechanism of antibiotic release from PMMA cement remains somewhat controversial, but the consensus would seem to be that the antibiotic is released in a biphasic manner, firstly eluded rapidly directly from the cement surface in the first hours to days, and subsequently from surface bleaching from a supposed network of cracks and voids in the bone cement over the course of weeks to months (Van de Belt et al., 2000). Many studies have suggested that up to 80 % of the antibiotic can get “locked away” in the PMMA cement, which is another downfall of this technique (Bertazzoni Minelli et al., 2011; Powles et al., 1998; Wroblewski et al., 1986). Both methods whether using cement or ceramic as a carrier for local antibiotic are off-label uses in the United States and require hand-mixing antibiotic into the coating material in the operating room.

There is a need for a novel carrier for the local release of antimicrobial agents to resolve the previously mentioned limitations. Calcium sulfate has emerged as a promising antibiotic carrier. It has been used as a bone void filler for a long time, and its popularity as a local antibiotic delivery system has recently grown in the treatment of musculoskeletal infections (Dreesmann, 1892; McPherson et al., 2013). Stimulan (Biocomposites Ltd., Keele, UK), which was introduced in 2000, is a synthetic crystallic semihydrate form of calcium sulfate. Just like with PMMA loaded with antibiotics, the early experience with Stimulan comes from the arthroplasty literature. McPherson et al. (2013) have reported on their clinical results using commercially pure synthetic antibiotic-loaded calcium sulfate dissolvable beads loaded with Vancomycin and Tobramycin in 250 cases of aseptic and septic revision total hip and knee arthroplasty, with good results (McPherson et al., 2013).

We have previously reported on our experience with antibiotic-loaded PMMA-coated IMN (PMMA-IMN) for limb salvage in septic complex lower extremity reconstruction (Makhdom et al., 2020). The goal of our study is to report on our recent experience with antibiotic-loaded calcium sulfate-coated interlocking intramedullary nails (CS-IMN) to prevent infection in high-risk patients and to eradicate infection when infection is confirmed. The second goal is to compare the results of our cohort where CS-IMN were used with curative intent with a prior cohort of patients treated with PMMA-IMN in terms of infection control rate, achievement of union or fusion, limb salvage rate, and overall complication rate.

## 2 Methods

After obtaining the institutional review board approval, we performed a retrospective review of the medical charts and radiographs for patients treated from January 2010 to August 2017 who underwent a limb salvage procedure with PMMA-IMN with a 2-year follow-up period and patients treated from May 2017 to June 2020 with the use of the novel CS-IMN for infection prevention or infection cure with a minimum of 6 months follow-up. We recorded patient demographics including indication for the surgery, anatomic affected site, pre-operative infecting organism (based on previous or intra-operative culture results), and host type (based on the Cierny–Mader classification (Cierny et al., 2003) (Tables 1–3). Outcomes include bony union, need for revision procedures, limb salvage rates, and recurrence of infection. Infection was either suspected or confirmed pre-operatively and postoperatively. In the CS-IMN cohort, some patients were considered to be high risk for infection and were treated prophylactically, while others were treated for a confirmed infection. Our indications for this technique to prevent infection were as follows: (1) one-stage conversions from external fixation to internal fixation, (2) patients with a history of recent infection in the planned operative site, (3) patients who had a history of external fixation for limb lengthening, given that the frames tended to be on for prolonged periods of time, and (4) patients with a history of an open fracture and resultant non-union, where there was an inherent high risk of occult septic non-union.

**Figure 1:**
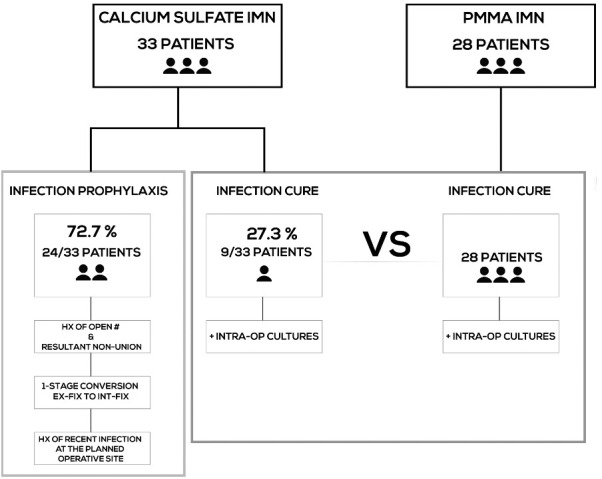
Methodology.

Another subset of patients in the CS-IMN cohort were being treated for a confirmed active infection, which was confirmed with positive intra-operative cultures at the time of index surgery. This is the cohort that was compared to our prior cohort of patients treated for an active infection with PMMA-IMN (Fig. 1).

Our surgical technique was the same in both cohorts with regards to the treatment of chronic wounds and osseus nonunion sites which involved soft- and hard-tissue debridement in a systematic fashion down to visible bleeding bone as required. Any existing hardware was removed. Intra-operative cultures were obtained. In the CS-IMN patients, the antibiotic-loaded Stimulan paste was then prepared. In a mixing bowl, the following powdered dry ingredients were mixed until homogenous: three packs of Stimulan powder (20 g, powder contents of Stimulan Rapid Cure, Biocomposites Ltd, UK,) and the antibiotics, most commonly 3 g of Vancomycin hydrochloride powder and 1.2 g of Tobramycin sulfate powder. Three Stimulan rehydration solutions were then added, as well as 2 mL of sterile water or saline. The content of the bowl was then whipped for 30 s to form a smooth wet paste with stiff peaks. The antibiotic calcium sulfate paste mixture was then placed in a Tumi syringe and connected to a 28 French chest tube. The mixture was then injected retrograde into the long bone canal. The IMN was then pushed in the canal displacing the paste which could be seen exiting previous bone holes (for example previous interlocking screw holes, or external fixator pin sites), ensuring that the entire intramedullary canal and to a certain extent adjacent soft tissues were coated with antibiotic.

In the PMMA group, standard locking intramedullary nails were used for all patients. The nails were coated with PMMA from Simplex (Stryker, Kalamazoo, MI) by filling a silicone tube with the antibiotic cement mixture and then inserting the IMN into the tube. The antibiotic regimen consisted of mixing 2 g of vancomycin and 2.4 g of tobramycin per bag (40 g) of simplex cement with tobramycin (1 g) (for a total dose of 3.4 g of tobramycin).

The cultures were followed in the postoperative period. In all cases of confirmed infection, under the guidance of an infectious disease specialist, the patients were treated with culture specific intravenous antibiotic therapy for a total duration of 6 weeks, followed by culture-specific oral suppressive antibiotics until bony union or joint fusion was achieved as per institutional protocol (Lam et al., 2019). Patients were monitored clinically and as required via laboratory testing during systemic antibiotic therapy. The infection was considered controlled by the absence of clinical infection (drainage, cellulitis, increased warmth) without suppressive antibiotic treatment. Patients were followed a minimum of 6 months in the CS-IMN cohort and over 2 years in the PMMA-IMN cohort. Laboratory infection values were not followed beyond the antibiotic therapy course regularly. Only if the infection was thought to be poorly controlled or recurring were the serum erythrocyte sedimentation rate and C-reactive protein obtained. Patients were considered to have bone union/fusion once they were pain free with full weight bearing and when there was union of a minimum of three out of four cortices on radiographs or CT scan. Clinical follow-up was every 3 months in the first year and every 6 months during the second year after surgery as required. Functional outcomes were assessed using the Association for the Study and Application of Methods of Ilizarov (ASAMI) criteria with the modification of adding amputation to the score as a “failure” (Paley et al., 1989). Fusion surgery patients could not qualify for an excellent score due to joint motion restriction.

**Table 1:** Patient demographics in the active infection treatment groups.

Patient demographics	CS-IMN for treatment of infection (9 patients)	PMMA-IMN for treatment of infection (28 patients)
Age	Mean 45 y.o. (range 31–73)	Mean 62 y.o. (range 22–88), p=0.0005
Sex	7 M, 2 F	16 M, 12 F, p=0.21
Type B Host	6/9 patients (66.7 %)	19/28 patients (67.9 %), p=0.77
Smoking	1/9 patients (11.1 %)	4/28 patients (14.3 %), p=0.44
DM2	0/9 patients	6/28 patients (21.4 %)
Mean follow-up (months)	28.3 (range 21.3–43.8)	40 (range 28–84)

**Table 2:** Anatomic location of septic complex lower extremity reconstruction in the active infection treatment groups.

Anatomic	CS-IMN For	PMMA-IMN for
location	treatment	treatment
	of infection	of infection
	(9 patients)	(28 patients)
Tibia	5	8
TTC fusion	2	6
Knee fusion	2	14

**Table 3:** Results of intra-operative cultures at our index surgery in the active infection treatment groups.

Cultured organisms	CS-IMN	PMMA-IMN
	(9 patients)	(28 patients)
	No. of patients	No. of patients
*Corynebacterium*	0	1
*E. Coli*	1	1
*Enterococcus*	0	1
MRSA	1	10
MRSE2	0	1
Polymicrobial	2	6
*Pseudomonas aeruginosa*	0	1
*S. epidermidis*	0	2
*S. lugdunensis*	1	1
VRE	0	2
MSSA	1	0
*Finegoldia magna*	1	0
Dermabacter species	1	0
MDR *E. faecium*	1	0
Negative cultures	0	2

## 3 Statistical analysis

Descriptive statistics in the form of means and ranges were utilized. The two-tailed Fisher exact test was used to compare categorical variables. Mann–Whitney U test was utilized to compare two independent means. The P value of <0.05 was considered statistically significant. The statistical package for the social sciences (Inc., Chicago, IL, USA) version 20.0 was utilized for the statistical work.

## 4 Results

A total of 33 patients were treated with CS-IMN and were eligible and included in the study. A total of 24/33 patients were treated for infection prevention due to high-risk clinical situations mentioned previously. There were 11 males and 13 females with a mean age of 52 years (range 24–74). A total of 14/24 patients were type B hosts (58.3 %) based on the Cierny–Mader classification; 7/24 patients were type-2 diabetics. These were patients with a high risk of infection, with 14/24 patients having a history of recent infection at the operative site, 9/24 patients treated for a presumed infected non-union, and 1/24 patients was a severely immunocompromised host. The mean follow-up period for this cohort was 30 months (range 6.5–63.8 months), with 7 patients having over 1-year follow-up and with 15 patients having more than 2-year follow-up. Eight patients were treated for femur non-union (33.3 %), six patients for tibial non-union (25 %), six patients for knee fusion (25 %), three patients for tibiotalocalcaneal fusion (12.5 %), and one patient for humeral non-union (4.2 %).

In this cohort, infection prevention was achieved in 24/24 patients (100 %). Bony union or fusion was achieved in 21/22 patients. Two patients were excluded from union/fusion analysis. One patient was pregnant at time of study, and therefore no radiographs could be performed to confirm union. One patient had a large cement spacer for bone defect, for which consolidation was not expected. In terms of the documented non-union: one patient was treated for a humerus fracture, and she was lost to follow-up, and non-union could not be excluded based of telephone follow-up.

**Table 4:** Primary outcomes for active infection CS-IMN versus PMMA-IMN.

Primary outcomes	CS-IMN for treatment of infection (8 patients)	PMMA-IMN for treatment of infection (28 patients)
Infection eradication	6/8 patients (77.8 %)	21/26 patients (80 %), p=0.42
Bone union/fusion	8/8 patients (100 %)	21/24 patients (87.5 %)
Limb salvage rate	8/8 patients (100 %)	25/28 patients (89 %)
Mean follow-up	28.3 months (range 21.3–43.8)	40 months (range 28–84)

**Table 5:** Functional ASAMI scores for patients treated for infection prevention and eradication with CS-IMN and for patients treated for infection eradication with PMMA-IMN.

Treatment group	Functional ASAMI score CS-IMN				
	Excellent	Good	Fair	Poor	Failure
Femur non-union ( N=8 )	6	2	0	0	0
Knee fusion ( N=8 )	0	7	1	0	0
Tibial non-union ( N=12 )	4	7	1	0	0
Ankle fusion ( N=4 )	0	2	1	0	0
Humerus non-union ( N=1 )	0	0	1	0	0
Treatment group	Functional ASAMI score PMMA-IMN				
	Excellent	Good	Fair	Poor	Failure
Knee fusion ( N=14 )	0	6	5	0	3
Tibial septic nonunion ( N=8 )	2	5	1	0	0
Ankle fusion ( N=6 )	0	3	3	0	0

A total of nine patients were treated with CS-IMN for active infection with curative intent. These were patients with positive intra-operative cultures. These patients were compared to our previous cohort of 28 patients treated with PMMA-IMN (Tables 1–3). The patients in the PMMA-IMN group were found to be statistically significantly older than in the CS-IMN group, otherwise the groups were well matched (Table 1). The mean follow-up in the CS-IMN group was 28.3 months (range 21.3–43.8 months) versus 40 months (range 28–84 months) in the PMMA-IMN group. The anatomic location was also recorded for both cohorts, with tibial non-unions being the most commonly treated pathology in the CS-IMN and knee fusions in the PMMA-IMN group (Table 2). The most common infective organism in the PMMA-IMN cohort was MRSA, while there was a wide range of cultured organisms in the CS-IMN group (Table 3).

In patients treated for active infection, one patient was removed from analysis on the CS-IMN group due to active intravenous drug use and loss to follow-up. The rate of infection eradication was 77.8 % ( 6/8 patients) in the CS-IMN cohort, as compared to 80 % ( 21/26 patients) in the PMMA-IMN cohort ( p=0.42 ). Bony union or fusion was achieved in 100 % ( 8/8 patient) in the CS-IMN group versus 87.5 % of patients in the PMMA-IMN group. Limb salvage rate was 100 % ( 8/8 patients) in the CS-IMN group versus 89 % in the PMMA-IMN group (Table 4).

In those patients who did experience recurrence of infection in the CS-IMN group, one septic knee fusion patient underwent repeat irrigation and debridement, with cultures positive for *Staphylococcus aureus*. The patient underwent exchange nailing with repeat antibiotic-loaded calcium sulfate-coated locked intramedullary nailing. The patient did not have infection recurrence at final follow-up. One patient was treated for septic tibia non-union. This patient underwent repeat irrigation and debridement, exchange nailing with repeat antibiotic-loaded calcium sulfate-coated locked intramedullary nailing. The patient had no infection recurrence at final follow-up.

As described in our previous paper, in the PMMA-IM nail cohort, one tibial septic non-union patient remained on chronic antibiotic suppression, and one underwent segmental resection and bone transport. Three septic knee fusion patients were treated with above-knee amputation for chronic infection and non-union. It has been previously described by our principal investigator that septic knee fusions are a very difficult problem to treat. Functional ASAMI scores indicated that most patients treated with CS-IMN and PMMA-IMN had good to excellent results (Table 5).

## 5 Discussion

The role of CS-IMN has emerged as a promising technique for prophylaxis and treatment of difficult long-bone infections. We have found CS-IMN easy to perform making it ideal for cases with significant risk for infection or in cases where infection is suspected. In these patients, CS-IMN were 100 % effective at infection prevention even in the presence of poor hosts ( 14/24 patients Cierny–Mader type B hosts). This may be a promising technique that could also be used prophylactically at index surgery for open tibial fractures, which are notoriously at high risk of septic non-union (Fuchs et al., 2011; Schmidmaier et al., 2006).

In patients where infection was confirmed and our intent was curative, CS-IMN were found to be non-inferior to PMMA-IMN for infection eradication. The cohort studies were small, and the data are only suggestive of non-inferiority at this time. In the patients initially treated with CS-IMN where infection recurred after our index surgery, revision surgery was performed by repeating another antibiotic-loaded calcium sulfate interlocked nail and the infection was eradicated. Based on this preliminary data, our center has completely converted to antibiotic-loaded calcium-coated interlocked intramedullary nails and stopped using antibiotic-loaded PMMA-coated implants.

Limitations of this study include a very small sample size, its retrospective nature, and shorter follow-up period in the CS-IMN group. This is a pilot study aiming to report on our preliminary data. There is no cost analysis on the two techniques. Calcium sulfate product raises the cost of surgery more so than PMMA use. However, PMMA coating is labor intensive and increases operative time, therefore raising the cost of surgery as well. Cases where an internal lengthening or compression implant is used cannot be used with PMMA. If the CS reduces the risk of infection and prevents additional surgery, then the minimal cost increase for using CS will be offset by the major savings accrued in avoiding more surgery. We plan to re-analyze the CS-IMN group in the future adding additional cases. The strength of this report is to help guide surgeons who are using similar methods haphazardly to create a uniform approach that can be analyzed when enough cases have been done. This report shows the method reported appears to be safe and effective.

In conclusion, the use of dissolving local antibiotic delivery systems will improve our workflow, reduce operative time, and improve infection control in orthopedic limb deformity and trauma surgery. Antibiotic-loaded calcium sulfate-coated interlocked intramedullary nails are a promising novel technique.

## 6 Level of evidence

Level III

## Data Availability

Data are available upon request.
